# The impact of levothyroxine therapy on pregnancy and neonatal outcomes in euthyroid pregnant women with thyroid autoimmunity: A systematic review, meta-analysis and trial sequential analysis

**DOI:** 10.3389/fphar.2023.1054935

**Published:** 2023-03-02

**Authors:** Jingjing Chen, Xue-Feng Jiao, Li Zhang, Miao Zhang, Linan Zeng, Dan Liu, Hailong Li, Kun Zou, Qiang Wei, Lingli Zhang

**Affiliations:** ^1^ Department of Pharmacy, West China Second University Hospital, Sichuan University, Chengdu, China; ^2^ Evidence-Based Pharmacy Center, West China Second University Hospital, Sichuan University, Chengdu, China; ^3^ NMPA Key Laboratory for Technical Research on Drug Products *In Vitro* and *In Vivo* Correlation, Chengdu, China; ^4^ Key Laboratory of Birth Defects and Related Diseases of Women and Children, Sichuan University, Ministry of Education, Chengdu, China; ^5^ Department of Obstetrics and Gynecology, West China Second University Hospital, Sichuan University, Chengdu, China; ^6^ West China School of Pharmacy, Sichuan University, Chengdu, China; ^7^ Chinese Evidence-based Medicine Center, West China Hospital, Sichuan University, Chengdu, China

**Keywords:** levothyroxine, pregnancy outcomes, neonatal outcomes, euthyroid pregnant women, thyroid autoimmunity

## Abstract

**Background:** At present, only one systematic review has investigated the effect of levothyroxine (LT4) in the treatment of euthyroid pregnant women with thyroid autoimmunity, but some problems [such as merging different types of research for meta-analysis, lacking neonatal outcomes, and so on] exist in this study, satisfactory results can not be provided. So, this systematic review was performed to investigate the effect of LT4 in euthyroid pregnant women with thyroid autoimmunity, in the hope of providing more comprehensive evidence for clinical use.

**Methods:** Medline (Ovid), Embase (Ovid), and Cochrane Central Register of Controlled Trials were electronically searched from database inception to March 2022. We included cohort studies and RCTs that evaluated the impact of LT4 therapy on pregnancy and neonatal outcomes in euthyroid pregnant women with thyroid autoimmunity. Meta-analyses of different types of studies were performed separately, and meta-analyses were further performed by only including researches with low and moderate risk of bias. We used the Grading of Recommendations, Assessment, Development and Evaluations (GRADE) approach to evaluate the quality of evidence, and used TSA to test the sufficiency of the evidence.

**Results:** Finally, 2,901 euthyroid pregnant women with thyroid autoimmunity in six RCTs and five cohort studies were included. In all outcomes, no statistically significant differences were found between LT4 group and control group, including miscarriage [RR = 0.85, 95%CI (0.69,1.05), *p* = 0.14, *I* ^2^ = 1%], preterm birth [RR = 0.80, 95%CI (0.59,1.08), *p* = 0.14, I2 = 0%], preeclampsia [RR = 0.68, 95%CI (0.12, 3.91), *p* = 0.66, *I* ^2^ = 0%], placenta abruption [Peto’ OR = 0.14, 95%CI (0.00, 6.94), *p* = 0.32, *I* ^2^ = 0%], birth weight [MD = -36.00, 95%CI (-170.41, 98.41), *p* = 0.60, *I* ^2^ = 0%], gestational age at delivery [MD = -0.10, 95%CI (-0.61, 0.41), *p* = 0.70, *I* ^2^ = 0%] and neonatal admission [RR = 1.33, 95%CI (0.21, 8.58), *p* = 0.76, *I* ^2^ = 0%]. The results for all outcomes were insufficient and inconclusive as demonstrated by TSA. The GRADE assessments showed that the quality of evidence of 4 outcomes (miscarriage, preterm birth, birth weight and gestational age at delivery) were moderate, and 3 outcomes (preeclampsia, placenta abruption and neonatal admission) were low or very low.

**Conclusion:** For pregnancy and neonatal outcomes in euthyroid pregnant women with thyroid autoimmunity, we did not find benefit of LT4 treatment in this study.

**Systematic Review Registration**: https://www.crd.york.ac.uk/prospero/display_record.php?ID=CRD42022346745, identifier CRD42022346745.

## 1 Introduction

Thyroid autoimmunity is characterized by elevated thyroid peroxidase antibody (TPOAb) and/or thyroglobulin antibody (TgAb) and found in 2%–17% of pregnant women (Benhadi et al., 2007; Pearce et al., 2008; Abbassi-Ghanavati et al., 2010; Ashoor et al., 2010; Moreno-Reyes et al., 2013). According to the 2017 American Thyroid Association (ATA) guideline, TPOAb-positive pregnant women with 2.5–4.0 mIU/L thyroid-stimulating hormone (TSH) in early pregnancy are considered as euthyroid pregnant women with thyroid autoimmunity (Alexander et al., 2017). Currently, levothyroxine (LT4) is widely used in these women. However, in this issue, the recommendations provided in different guidelines differ markedly. For example, LT4 is recommended for pregnant women with TSH 2.5–4.0 mIU/L and TPOAb in early pregnancy in the 2019 Chinese Medical Association (CMA) guideline (Association CM, 2019), but LT4 is weakly recommended for them in the 2017 American Thyroid Association (ATA) guideline; What’s more, LT4 therapy is not recommended for pregnant women with TSH 2.5–4.0 mIU/L and TPOAb in early pregnancy in the 2020 American College of Obstetricians and Gynecologists (ACOG) guideline (Autho r Anonymous, 2020).

At present, only one meta-analysis explored the impact of LT4 treatment in pregnant euthyroid women with thyroid autoimmunity (Sun et al., 2020). However, a series of problems exist in this study. Firstly, data of different types of studies (cohort studies and RCTs) were synthesized in one meta-analysis (Cochrane Training, 2022). Because of methodological heterogeneity, it may result in incorrect results (Cochrane Training, 2022). Secondly, only pregnancy outcomes were assessed in this study, and neonatal outcomes were not assessed. Thirdly, in this study, neither the GRADE method nor TSA were performed to evaluate the quality of the current evidence and to test whether the current studies had enough statistical power to reach a firm conclusion. Both of them are important for high-quality systematic review and meta-analysis.

So, we performed a systematic review, meta-analysis and TSA to evaluate the effect of LT4 treatment on pregnancy and neonatal outcomes in euthyroid pregnant women with thyroid autoimmunity, in the hope of providing more comprehensive and credible evidence for clinical use.

## 2 Materials and methods

This study was reported in line with the PRISMA (Moher et al., 2009).

### 2.1 Inclusion criteria

#### 2.1.1 Participants

Euthyroid pregnant women with positive TPOAb and/or TgAb. The diagnosis was based on the diagnostic criteria in the 2017 ATA guideline.

#### 2.1.2 Interventions and controls

LT4 compared with no treatment or placebo. Dosages and treatment duration were not limited.

#### 2.1.3 Outcomes

Primary outcomes: 1) pregnancy outcomes: miscarriage, preterm birth, preeclampsia.

Secondary outcomes: 1) pregnancy outcomes: placenta abruption; 2) neonatal outcomes: gestational age at delivery, birth weight, neonatal admission.

#### 2.1.4 Types of studies

RCTs and cohort studies were enrolled in this systematic review.

### 2.2 Exclusion criteria

Full-text not available; outcome data not available; duplicate publications.

### 2.3 Data sources and retrieval strategy

We searched databases including Medline (Ovid), EMbase (Ovid) and the Cochrane Central Register of Controlled Trials from inception to March 2022. The references of the included studies were also checked for additional studies. The retrieval strategy of Medline (Ovid) is shown in Supplementary Table S1.

### 2.4 Study selection and data extraction

The titles and abstracts of all studies were screened by 2 researchers independently to determine which studies were potentially relevant. Then, two reviewers independently assessed the full texts. If there were any disagreements, a third researcher resolved it.

2 researchers independently extracted the data using a form. The extracted information included basic information of the study, basic information of the pregnant women, interventions and comparations, sample sizes and outcome data.

### 2.5 Assessment of risk of bias

2 researchers assessed the risk of bias, and we resolved the disagreements were by discussion with a third researcher.

For RCTs, we used the RoB2 tool recommended by the Cochrane Handbook to evaluated the risk of bias (Sterne et al., 2019) and this tool provided a bias summary for every study rated as “low risk of bias”, “some concerns (moderate risk of bias)” or “high risk of bias”.

For cohort studies, we used the Newcastle-Ottawa Quality Assessment Scale (NOS) to evaluated the risk of bias (Stang, 2010). 3 aspects were included in this tool and a score of 7–9 was rated as “low risk of bias”, 4–6 was rated as “moderate risk of bias”, and 0–3 was rated as “high risk of bias”.

### 2.6 Quality of evidence

For each outcome, the GRADE was used in evaluating the quality of the evidence (Atkins et al., 2004). 5 aspects (risk of bias, reporting biases, imprecisions, inconsistencies and indirectness) were considered and the quality of evidence was classified into 4 levels: high, moderate, low and very low (Balshem et al., 2011).

### 2.7 Statistical analysis

Meta-analyses were performed by RevMan 5.4. For dichotomous data, we calculated the relative risk (RR) or odds ratio (OR) with 95% confidence interval (CI) to evaluate the effect measure, and for continuous data, we calculated the mean difference (MD) with 95% CI to evaluate the effect measure. Besides, we calculated the Peto’s OR with 95% CI for dichotomous outcomes with zero-events. We assessed heterogeneity by I-squared (*I* ^*2*^) test. If heterogeneity was acceptable (*I* ^*2*^ ≤ 50%), we used a fixed effect model, otherwise, we used a random effect model.

#### 2.7.1 Meta-analysis

We separately performed meta-analyses of different types of studies. We also performed meta-analyses by only including researches with low and moderate risk of bias to evaluate the influence of poor-quality studies. For each outcome, we drew conclusions by following the rules below (Guyatt et al., 2011):

If the evidence quality of RCTs was higher than the evidence quality of cohort studies, conclusion was drew based on the meta-analysis result of RCTs. Otherwise, conclusion was drew based on the meta-analysis result of cohort studies. If there were no obvious difference in the meta-analysis result between all studies and studies with low and moderate risk of bias, conclusion was drew based on the result of all studies. If obvious difference exist between them, conclusion was drew based on the result of studies with low and moderate risk of bias.

#### 2.7.2 TSA analysis

We conducted TSA using the TSA viewer version 0.9.5.10 Beta to test whether the included studies had enough statistical power to reach a firm conclusion (Copenhagen Trial Unit, 2022).

## 3 Results

### 3.1 Results of search and study selection

**FIGURE 1:**
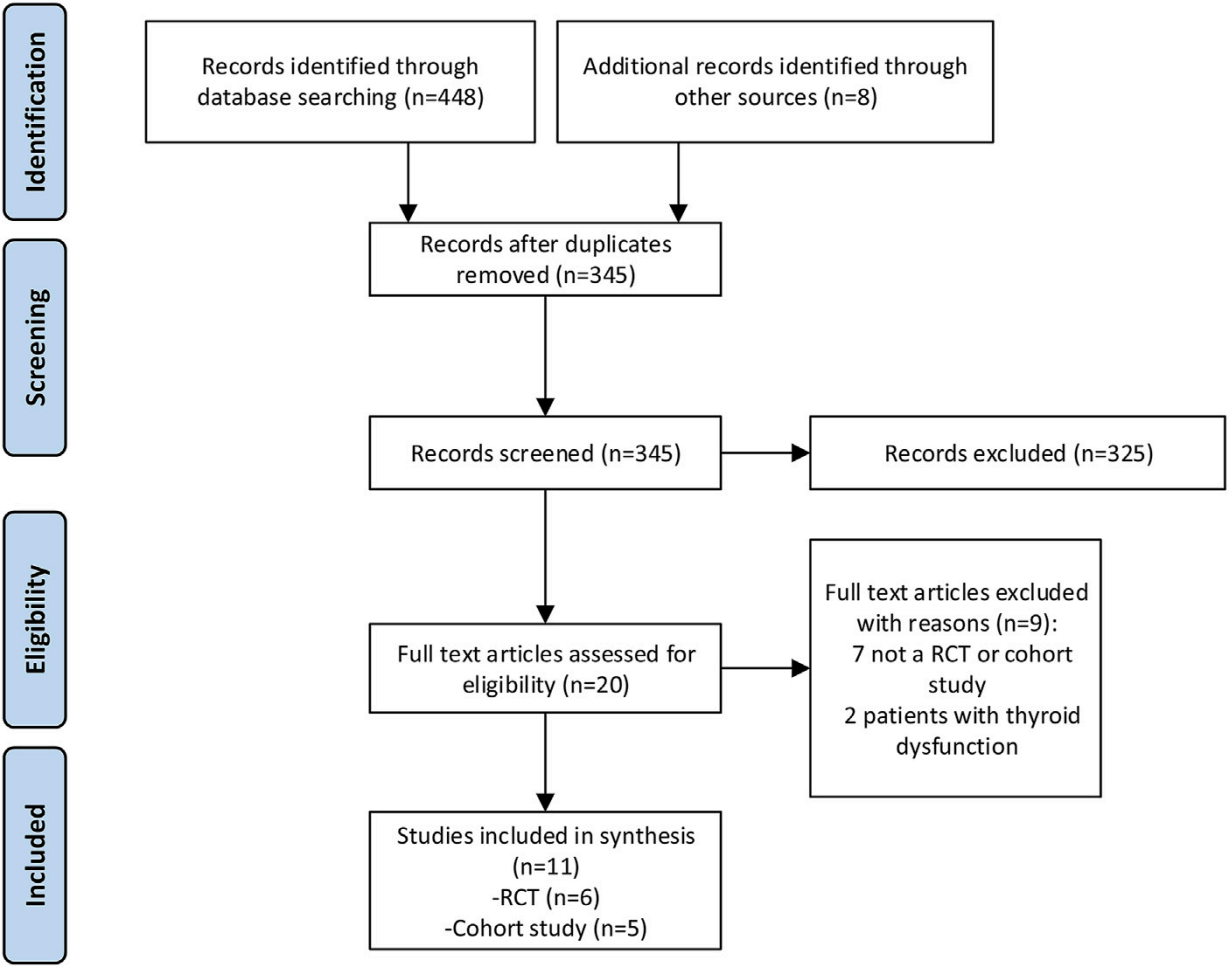
Study flow diagram.

In the initial search, we identified a total of 448 studies. Finally, 11 studies (6 RCTs and 5 cohort studies) met our inclusion criteria and were enrolled in this study (Figure 1).

### 3.2 Study characteristics

11 studies (Negro et al., 2005; Negro et al., 2006; Revelli et al., 2009; Lepoutre et al., 2012; Mosaddegh et al., 2012; Negro et al., 2016; Nazarpour et al., 2017; Wang et al., 2017; Dhillon-Smith et al., 2019; Dal Lago et al., 2021; Tsunemi et al., 2021) comprising 2,901 euthyroid pregnant women with thyroid autoimmunity were included. These studies took place in a variety of countries: 3 RCTs and 2 cohort studies in Italy, 1 RCT and 1 cohort study in Iran, 1 RCT in China, 1 RCT in United Kingdom, 1 cohort study in Japan and 1 cohort study in Belgium. Details of the included studies are summarized in Table 1.

**TABLE 1:** Characteristics of the included studies.

Study	Types of study	Country	Sample size		Age (year)		TSH (normal range; mIU/L)	Baseline TSH (median/mean, LT4/control)	Defintion of TPOAb positivity (IU/ml)
			LT4	Control	LT4	Control			
Negro 2005	RCT	Italy	36	36	29.2 ± 4.0	30.1 ± 5.0	0.27–4.2	1.9/1.7 mIU/L	>100
Negro 2006	RCT	Italy	57	58	30.0 ± 5.0	30.0 ± 6.0	0.27–4.2	1.6/1.7 mIU/L	>100
Negro 2016	RCT	Italy	198	195	28.9 ± 5.2	29.9 ± 5.1	0.5–2.5	1.4/1.4 mIU/L	>16
Nazarpour 2017	RCT	Iran	18	24	26.6 ± 5.8	27.0 ± 4.7	0.1–4	3.7/3.2 mIU/L	>50
Wang 2017	RCT	China	300	300	31.3 ± 3.9	31.7 ± 3.8	0.5–4.78	2.9/2.1 mIU/L	≥60
Dhillon-Smith 2019	RCT	United Kingdom	476	476	32.5 ± 4.9	32.7 ± 4.9	0.44–3.63	2.1/2.0 mIU/L	>99% concordance in the U.K. NEQAS IIA
Revelli 2009	Cohort study	Italy	55	38	35.1 ± 4.1	37.0 ± 3.5	NR	2.1/2.0 mIU/L	≥35
Lepoutre 2012	Cohort study	Belgium	49	47	31.5 ± 5.5	32.5 ± 5.3	0.2–3.5	NR mIU/L	≥9
Mohammad 2012	Cohort study	Iran	39	6	NR	NR	NR	NR mIU/L	>40
Alessandro 2021	Cohort study	Italy	227	230	36.3 ± 4.4	34.7 ± 4.4	0.4–2.5	1.7/1.4 mIU/L	unknown
Tsunemi A 2021	Cohort study	Japan	25	11	36.1 ± 5.3	36.8 ± 5.0	2.5-URL	3.4/3.3 mIU/L	≥16

RCT, randomized controlled trial; LT4, levothyroxine; TSH, thyroid-stimulating hormone; NR, not reported; URL, upper reference limit.

### 3.3 Assessment of risk of bias

The risk of bias assessments are shown in Supplementary Tables S2, S3. For RCTs, the following features were determined to be at high risk of bias: randomization process in 1 RCT (Nazarpour et al., 2017), deviations from intended interventions in 2 studies (Negro et al., 2006; Negro et al., 2016), measurement of outcomes in 2 studies (Negro et al., 2006; Negro et al., 2016). Overall, 3 RCTs were at a high risk of bias (Negro et al., 2006; Negro et al., 2016; Nazarpour et al., 2017), 2 RCTs had some concerns (moderate risk of bias) (Negro et al., 2005; Wang et al., 2017), and 1 RCT were at a low risk of bias (Dhillon-Smith et al., 2019). For cohort studies, 4 studies were at a high risk of bias (Revelli et al., 2009; Lepoutre et al., 2012; Mosaddegh et al., 2012; Dal Lago et al., 2021), and 1 study was at moderate risk of bias (Tsunemi et al., 2021).

### 3.4 Meta-analysis results

#### 3.4.1 Primary outcome

##### 3.4.1.1 Miscarriage

6 RCTs and 5 cohort studies reported miscarriage.

For RCTs, the results found no significant difference between LT4 group and control group in miscarriage [RR = 0.85, 95%CI (0.69,1.05), *p* = 0.14, *I* ^2^ = 1%] (Table 2). When only including researches with low and moderate risk of bias, there was still no obvious difference between two groups in miscarriage [RR = 0.91, 95%CI (0.71,1.17), *p* = 0.47, *I* ^2^ = 12%]. (Table 2).

**TABLE 2:** Results of meta-analysis for all outcomes.

Outcome	Included studies	Number of studies	Number of patients	MD/RR/OR	95% CI	*P*	*I* ^*2*^ (%)	Model
				/Peto’s OR				
Primary outcomes								
Miscarriage	all RCTs	5	1,313	0.85	0.69-1.05	0.14	1	Fix
	RCTs with low to moderate risk of bias	2	585	0.91	0.71-1.17	0.47	12	Fix
	all cohort study	5	397	0.12^a^	0.07-0.19	<0.001	62	Fix
	Cohort study with low to moderate risk of bias	1	19	1.5	0.12-18.36	0.75	0	Fix
Preterm birth	all RCTs	5	1,282	0.80	0.59-1.08	0.14	0	Fix
	RCTs with low to moderate risk of bias	1	540	0.85	0.53-1.36	0.49	0	Fix
	all Cohort study	2	134	0.95^a^	0.24-3.79	0.94	0	Fix
Preeclampsia	all RCTs	1	115	0.68	0.12-3.91	0.66	0	Fix
Secondary outcomes								
Placenta abruption	all RCTs	1	115	0.14^a^	0.00-6.94	0.32	0	Fix
	all cohort study	1	96	7.09^a^	0.14-357.80	0.33	0	Fix
Birth weight	all RCTs	1	375	−36.00	-170.41-98.41	0.60	0	Fix
Gestational age at delivery	all RCTs	1	374	−0.10	-0.61-0.41	0.70	0	Fix
Neonatal admission	all RCTs	1	42	1.33	0.21-8.58	0.76	0	Fix

^a^ Peto’s OR; RCTs, randomized controlled trials; MD, mean difference; OR, odds ratio; RR, relative risk; CI, confidence interval; *I* ^*2*^, statistical heterogeneity. According to the pre-defined rules, the meta-analysis results of all RCTs were used to draw conclusions for each outcome.

For cohort studies, the results of all studies showed that LT4 group compared with the control group had a lower risk of miscarriage [Peto’ OR = 0.12, 95%CI (0.07, 0.19), *p* < 0.01, *I* ^2^ = 62%] (Table 1). When we excluded studies with high risk of bias, no obvious difference between two groups in miscarriage were found [RR = 1.50, 95%CI (0.12,18.36), *p* = 0.75, *I* ^*2*^ = 0%]. (Table 2).

**FIGURE 2:**
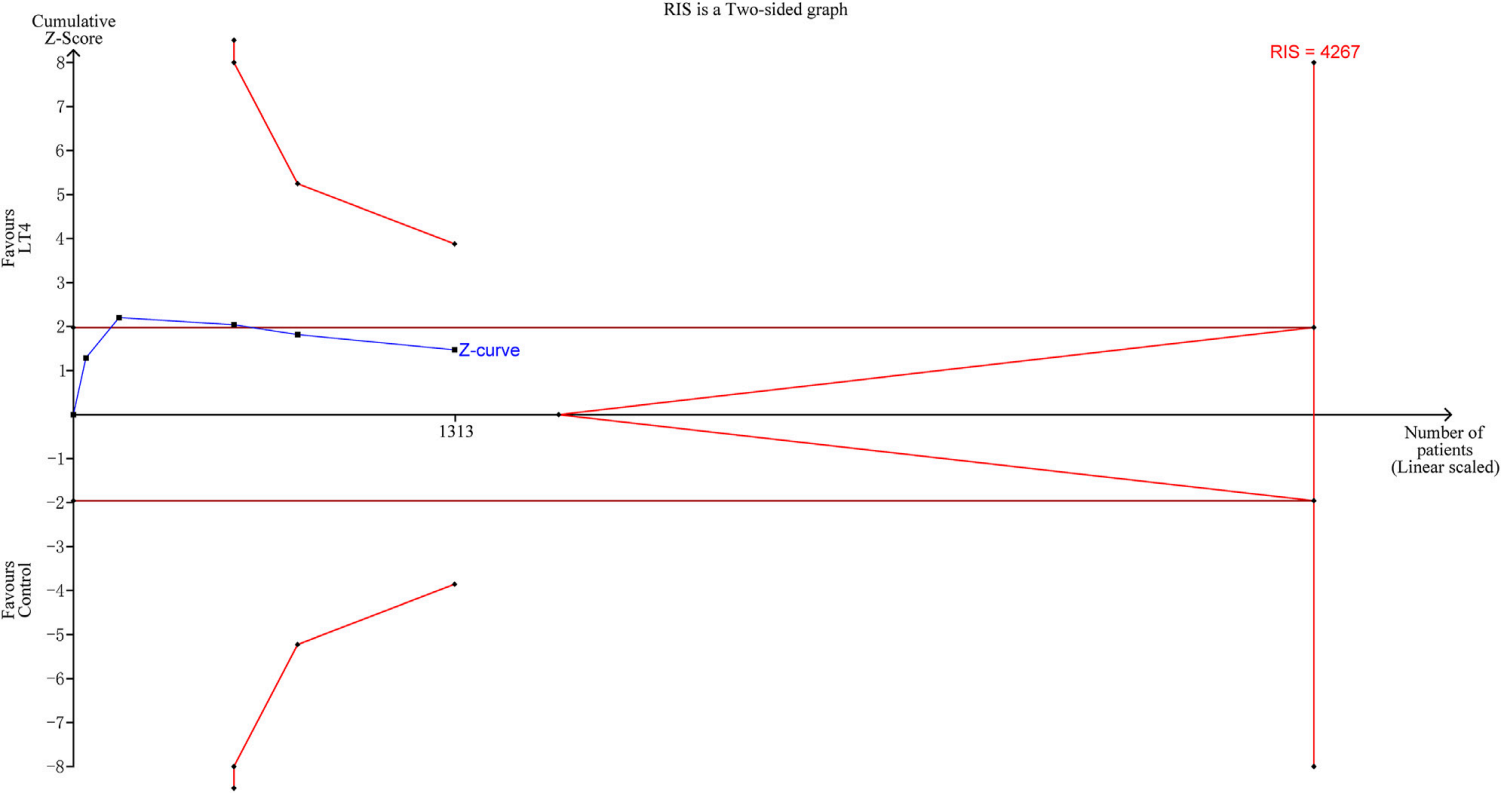
Trial sequential analysis of miscarriage. The risk of type I error was set at 5% with a power of 80%. The relative risk reduction (RRR) was set at 20%. The variance was calculated from the data obtained from the included trials.

TSA indicated that the cumulative information size (n = 1,313) was 31% of required information size (RIS) (n = 4,267). The cumulative Z curve did not cross the trial sequential monitoring boundary or the futility boundary, indicating that current evidence was insufficient and inconclusive. (Figure 2).

The GRADE assessment showed that the quality of evidence for miscarriage was moderate (Supplementary Table S4).

##### 3.4.1.2 Preterm birth

5 RCTs and 2 cohort studies reported preterm birth.

For RCTs, the results found no significant difference between LT4 group and control group in preterm birth [RR = 0.80, 95%CI (0.59,1.08), *p* = 0.14, *I* ^2^ = 0%]. When only including researches with low and moderate risk of bias, there was still no obvious difference between two groups in preterm birth [RR = 0.85, 95%CI (0.53,1.36), *p* = 0.49, *I* ^*2*^ = 0%]. (Table 2).

For cohort studies, the results found no significant difference between LT4 group and control group in preterm birth [Peto’ OR = 0.95, 95%CI (0.24, 3.79), *p* = 0.94, *I* ^2^ = 0%]. (Table 2).

**FIGURE 3:**
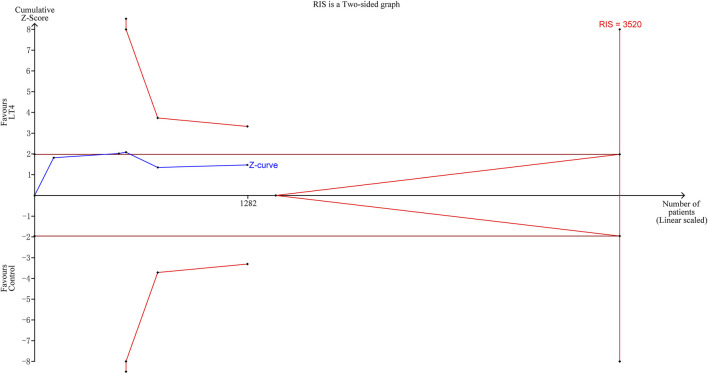
Trial sequential analysis of preterm birth. The risk of type I error was set at 5% with a power of 80%. The relative risk reduction (RRR) was set at 20%. The variance was calculated from the data obtained from the included trials.

TSA indicated that the cumulative information size (n = 1,282) was 36% of RIS (n = 3,520). The cumulative Z curve did not cross the trial sequential monitoring boundary or the futility boundary, indicating that current evidence was insufficient. (Figure 3).

The GRADE assessment showed that the quality of evidence for preterm birth was moderate. (Supplementary Table S4).

##### 3.4.1.3 Preeclampsia

A total of 1 RCT reported preeclampsia.

the results found no significant difference between LT4 group and control group in preeclampsia [RR = 0.68, 95%CI (0.12, 3.91), *p* = 0.66, *I* ^*2*^ = 0%]. (Table 2).

**FIGURE 4:**
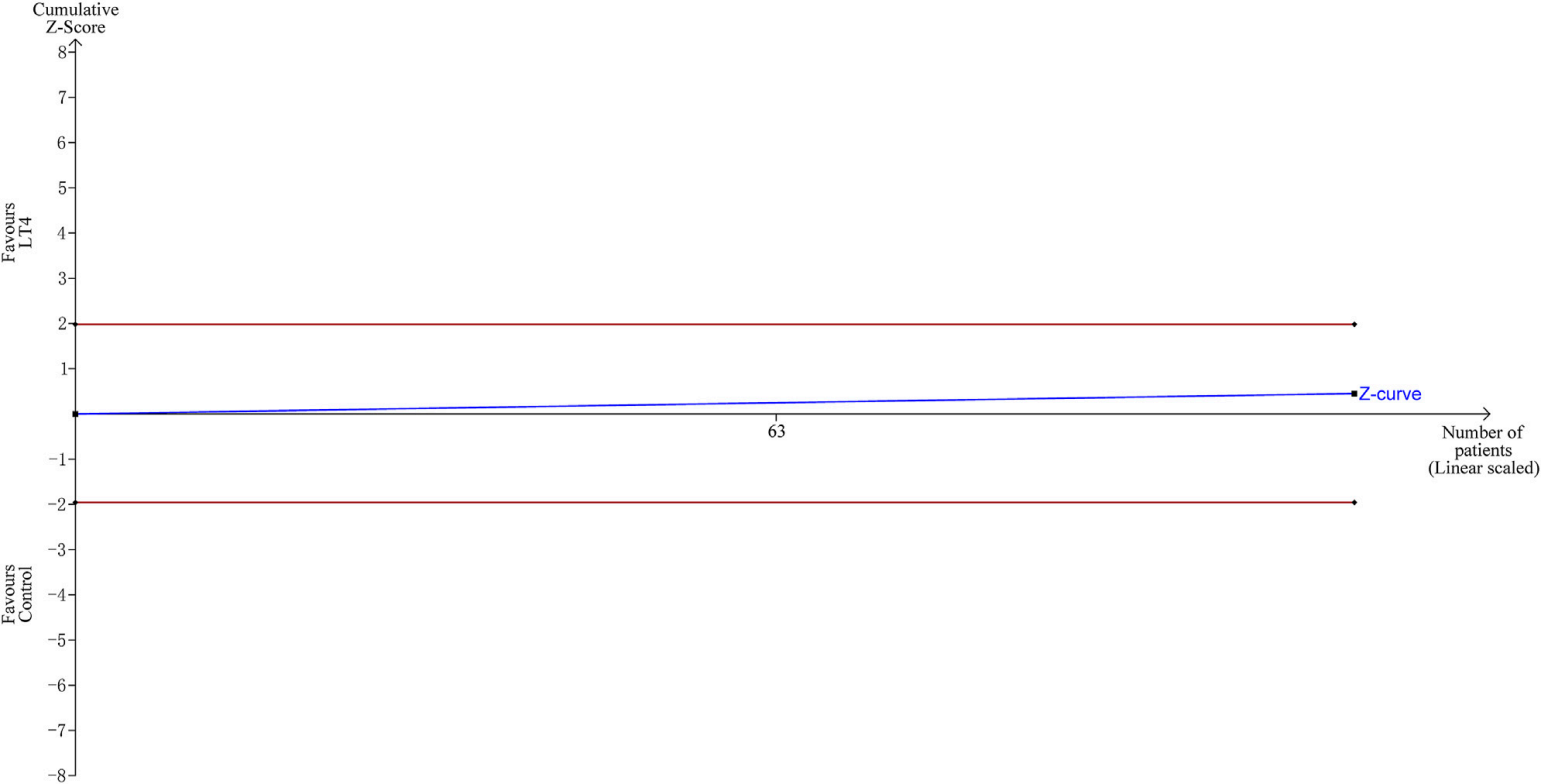
Trial sequential analysis of preeclampsia. The risk of type I error was set at 5% with a power of 80%. The relative risk reduction (RRR) was set at 20%. The variance was calculated from the data obtained from the included trials.

TSA indicated that the cumulative information size (n = 115) was 1% of RIS (n = 13493). The cumulative Z-curve did not cross the trial sequential monitoring boundary or the futility boundary, indicating that current evidence was insufficient and inconclusive. (Figure 4).

The GRADE assessment showed that the quality of evidence for preeclampsia was very low. (Supplementary Table S4).

#### 3.4.2 Secondary outcomes

The meta-analyses of RCTs found no significant difference between LT4 group and control group in placenta abruption [Peto’ OR = 0.14, 95%CI (0.00, 6.94), *p* = 0.32, *I* ^*2*^ = 0%], birth weight [MD = -36.00, 95%CI (-170.41, 98.41), *p* = 0.60, *I* ^*2*^ = 0%], gestational age at delivery [MD = -0.10, 95%CI (-0.61, 0.41), *p* = 0.70, *I* ^*2*^ = 0%] or neonatal admission [RR = 1.33, 95%CI (0.21, 8.58), *p* = 0.76, *I* ^*2*^ = 0%]. The meta-analyses of cohort studies also found no significant difference between two groups in placenta abruption [Peto’ OR = 7.09, 95%CI (0.14, 357.80), *p* = 0.33, *I* ^*2*^ = 0%]. (Table 2).

TSA indicated that the current evidences for placenta abruption, birth weight, gestational age at delivery and neonatal admission were insufficient and inconclusive. (Supplementary Figures S1–S4).

The GRADE assessment showed that for birth weight and gestational age at delivery, the quality of evidence was moderate; for placenta abruption and neonatal admission, the quality of evidence was very low. (Supplementary Table S4).

## 4 Discussion

### 4.1 Principle findings

To our knowledge, this study firstly explored the impact of LT4 in euthyroid pregnant women with thyroid autoimmunity using the TSA method and the GRADE approach, and is the most comprehensive systematic review assessing this effect. Our study did not find obvious differences between two groups (LT4 vs control group) in all pregnancy and neonatal outcomes (miscarriage, preterm birth, preeclampsia, placenta abruption, birth weight, gestational age at delivery and neonatal admission). TSA indicated that the result for all primary and secondary outcomes were insufficient and inconclusive. The GRADE assessments indicated that the quality of evidences for 4 outcomes (miscarriage, preterm birth, birth weight and gestational age at delivery) were moderate, and the quality of evidences for the other 3 outcomes (preeclampsia, placenta abruption and neonatal admission) were low or very low quality.

### 4.2 Compared with previous studies

Many systematic reviews have assessed the role of LT4 treatment in pregnant women with thyroid autoimmunity (Rao et al., 2019; Sun et al., 2020; Wang et al., 2020; Lau et al., 2021). However, these studies synthesized the data of both pregnant women with subclinical hypothyroidism and euthyroid pregnant women. Rao et al., in 2019 (Rao et al., 2019) enrolled 5 RCT and 2 cohort study of women with thyroid autoimmunity and found that LT4 significantly in reduced the risk of pregnancy loss. However, patients with subclinical hypothyroidism were also included and an important RCT published in 2019 (Dhillon-Smith et al., 2019) were not enrolled. This RCT assessed the impact of LT4 in euthyroid women who had thyroid autoimmunity and a history of miscarriage or infertility, and this study reached an unexpected conclusion that LT4 did not reduce the adverse outcomes (Dhillon-Smith et al., 2019). Wang et al. (Wang et al., 2020)in 2020 assessed the impact of LT4 in pregnant patients with thyroid autoantibodies and 6 RCT were included with a conclusion that LT4 showed no obvious benefit for the target patients. Lorraine Lau et al. (Lau et al., 2021) in 2021 also assessed the role of LT4 in pregnant women with thyroid autoimmunity and did not found difference between LT4 and control groups. The two researches also enrolled patients with subclinical hypothyroidism. Only one study (Sun et al., 2020) restricted the population to euthyroid pregnant women with thyroid autoimmunity. However, a series of problems exist in this study, including synthesizing different types of studies (RCTs and cohort studies) for meta-analysis, lacking neonatal outcomes, lacking TSA analysis, lacking evaluation of the quality of evidence. Thus, the above-mentioned systematic reviews cannot provide guidance on the need for LT4 therapy in euthyroid pregnant women with thyroid autoimmunity. Our study resolved these problems and provided more comprehensive and accurate results than the above-mentioned study.

### 4.3 Explanation of unexpected findings

Although significant differences between LT4 and control groups were not found in our study, trends toward decreased risks of some outcomes (such as miscarriage and preterm birth) exist. Moreover, results of TSA indicated that statistical power of the current RCTs for these outcomes were not enough to reach firm conclusions. So, in our study, these negative results might be induced by the relatively small sample size and be altered by future high-quality studies.

### 4.4 Implications for clinical recommendations

Currently, LT4 is frequently applied in euthyroid pregnant women with thyroid autoimmunity, particularly in China. Besides, the two most widely used guidelines (the 2017 ATA guideline (Alexander et al., 2017) and the 2019 CMA guideline (Association CM, 2019)) both recommend LT4 treatment for euthyroid pregnant women with thyroid autoimmunity, although the strength of recommendations differs among these two guidelines. However, our study did not find evidence of benefit for LT4 therapy in euthyroid pregnant women with thyroid autoimmunity.

Several studies have demonstrated that the presence of TPOAb in euthyroid pregnant women is associated with an increased risk of miscarriage, that is 2–3 times higher than in pregnant women without TPOAb (Chen and Hu, 2011; Thangaratinam et al., 2011). Most clinical studies mainly focused on the adverse impact of low thyroid hormone level on pregnant women, but some studies also demonstrated the adverse impact of high thyroid hormone level. Animal studies have found that high level of thyroid hormone adversely affect brain development (Marta et al., 1998), and a large prospective cohort study in 2016 investigated the association of maternal thyroid function with child intelligence quotient (IQ) and brain morphology (Korevaar et al., 2016). This cohort study found that both low and high maternal free thyroxine concentrations during pregnancy were associated with lower child IQ and lower grey matter and cortex volume. When excluding women with overt hypothyroidism and overt hyperthyroidism, all associations did not change, indicating that LT4 treatment during pregnancy may increase the potential risk of adverse offspring outcomes, regardless of the thyroid function.

Based on our results and the potential adverse impact of LT4 on offspring outcomes, the widespread use of LT4 in euthyroid pregnant women with thyroid autoimmunity may not be appropriate, and some revision may be required for the recommendations of these two guidelines. Moreover, the 2020 ACOG guideline does not recommend LT4 therapy for euthyroid pregnant women with thyroid autoimmunity (Autho r Anonymous, 2020), which is supported by our results and need to be taken into consideration in future clinical practice. In addition, according to our TSA results, large sample size RCTs of high quality are still warranted in the future to reach a firm conclusion. So, pros and cons of LT4 must be fully weight before making the decision.

### 4.5 Strengths and limitations

Our study has many strengths. Firstly, we restricted study population to euthyroid pregnant women with thyroid autoimmunity only and assessed both pregnancy and neonatal outcomes, which could precisely and comprehensively answer the clinical question of whether LT4 therapy is required in euthyroid pregnant women with thyroid autoimmunity. Secondly, our study used the well recommended statistical method to deal with zero-events in meta-analysis (Sweeting et al., 2004; Xu et al., 2021), and performed TSA to test whether the current evidence was sufficient, which further ensured the reliability of our study.

Our study also has some limitations. Firstly, the number of included researches and the total sample size were relatively limited, which induced inadequate statistical power to draw firm conclusions for some outcomes. Secondly, we failed to evaluate the impact of LT4 therapy on childhood outcomes due to lack of information from the original studies. Thirdly, the risk of publication bias cannot be ruled out due to the limited number of enrolled researches.

## 5 Conclusion

Our study found no evidence of benefit of LT4 therapy on pregnancy and neonatal outcomes in euthyroid pregnant women with thyroid autoimmunity. This finding do not support LT4 therapy for euthyroid pregnant women with thyroid autoimmunity. Further and large sample size RCTs of high quality are still needed to clarify this issue.

## Data Availability

The raw data supporting the conclusions of this article will be made available by the authors, without undue reservation.
