# Supplementary material for: The impact of levothyroxine therapy on pregnancy and neonatal outcomes in euthyroid pregnant women with thyroid autoimmunity: A systematic review, meta-analysis and trial sequential analysis
Source: Front Pharmacol. 2023 Mar 2;14:1054935. doi: 10.3389/fphar.2023.1054935 (PMC10018182; doi:10.3389/fphar.2023.1054935)
Supplement: Supplementary file 1 [file Table1.DOCX]

Supplementary Material

# Supplementary Figures and Tables

## Supplementary Figures


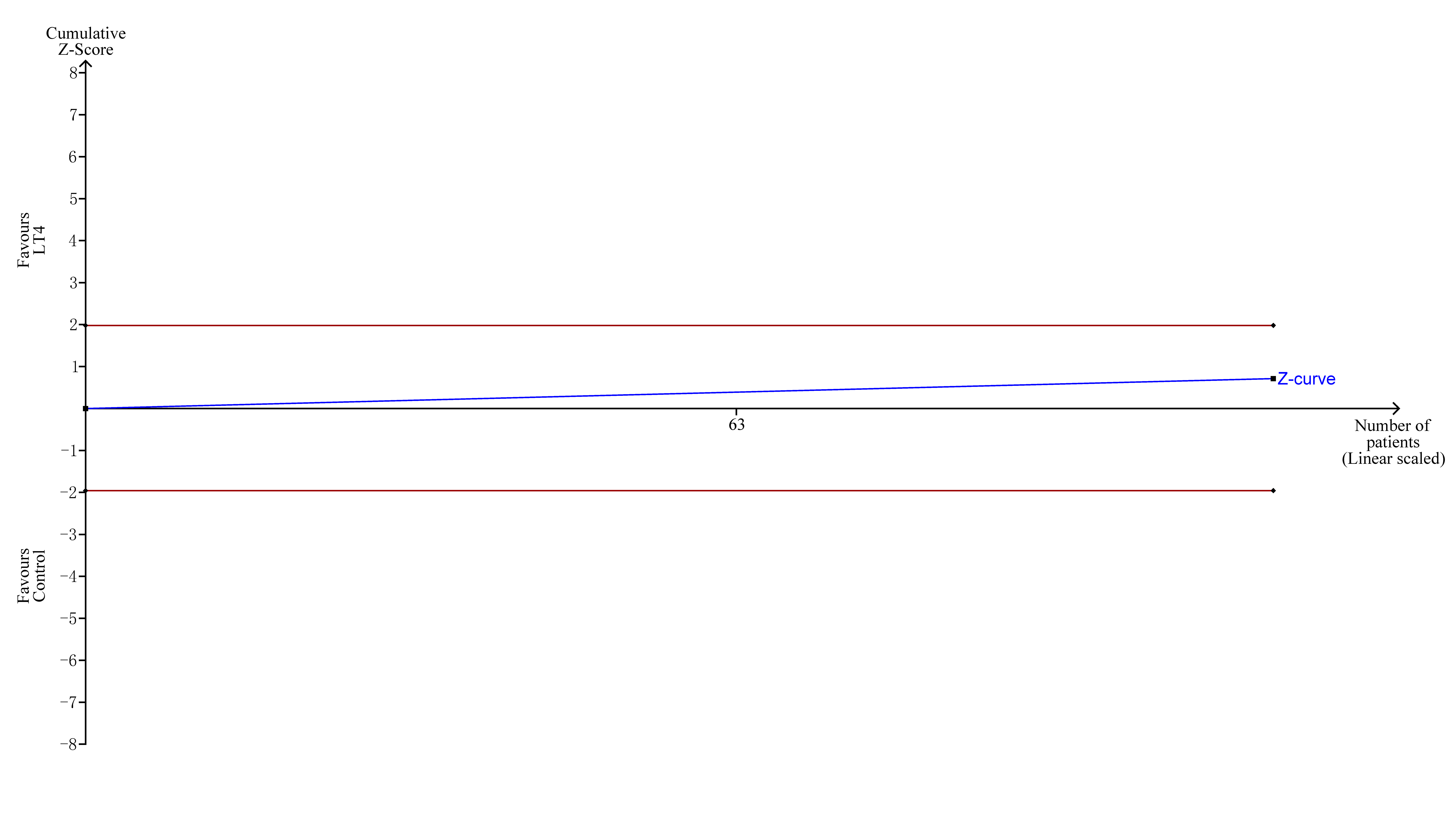


**Supplementary** **Figure 1. Trial sequential analysis of placenta abruption.** The risk of type Ⅰ error was set at 5% with a power of 80%. The variance was calculated from the data obtained from the included trials. The relative risk reduction (RRR) was set at 20%.


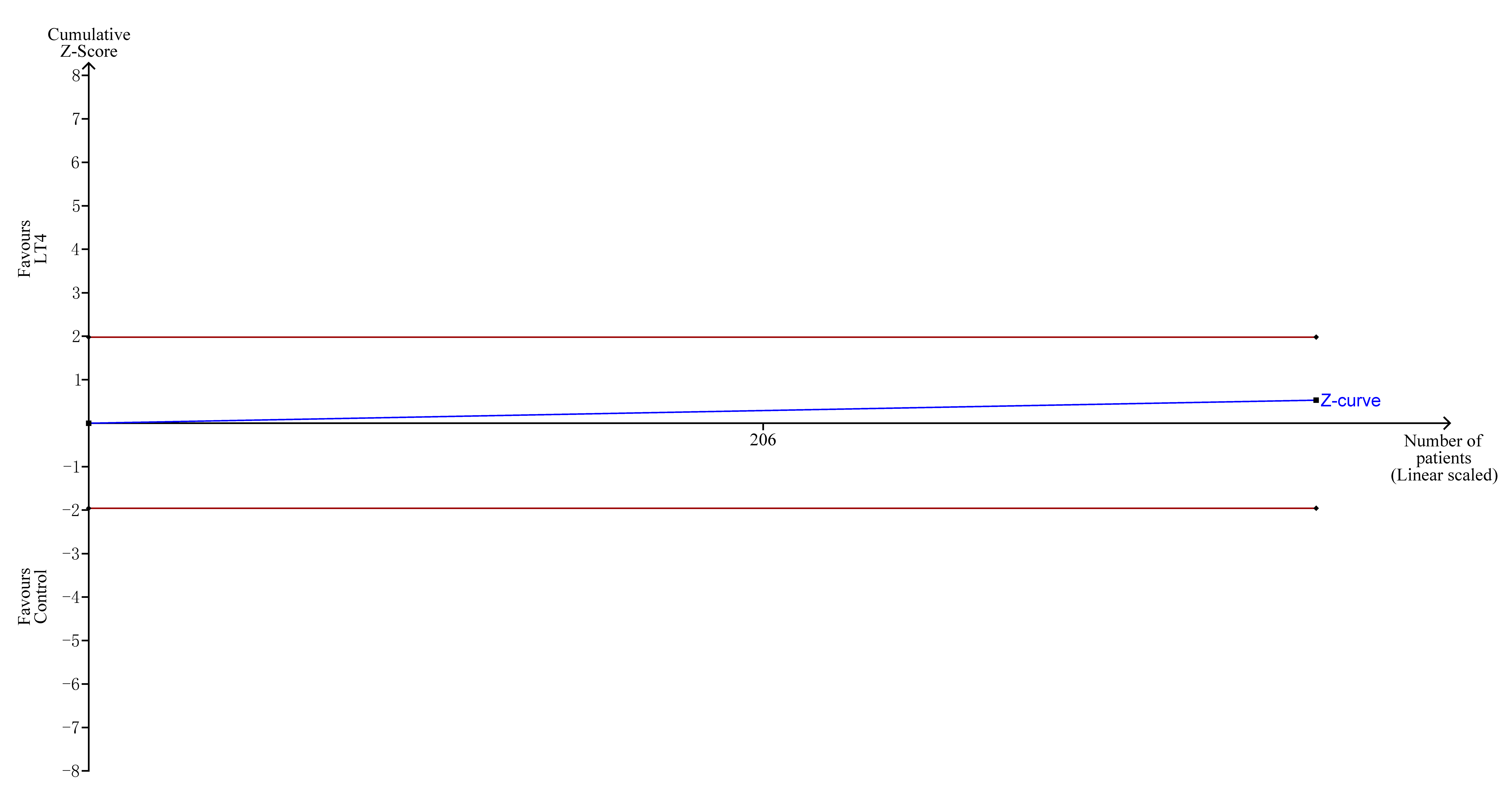


**Supplementary Figure 2. Trial sequential analysis of birth weight.** The risk of type Ⅰ error was set at 5% with a power of 80%. The variance was calculated from the data obtained from the included trials.


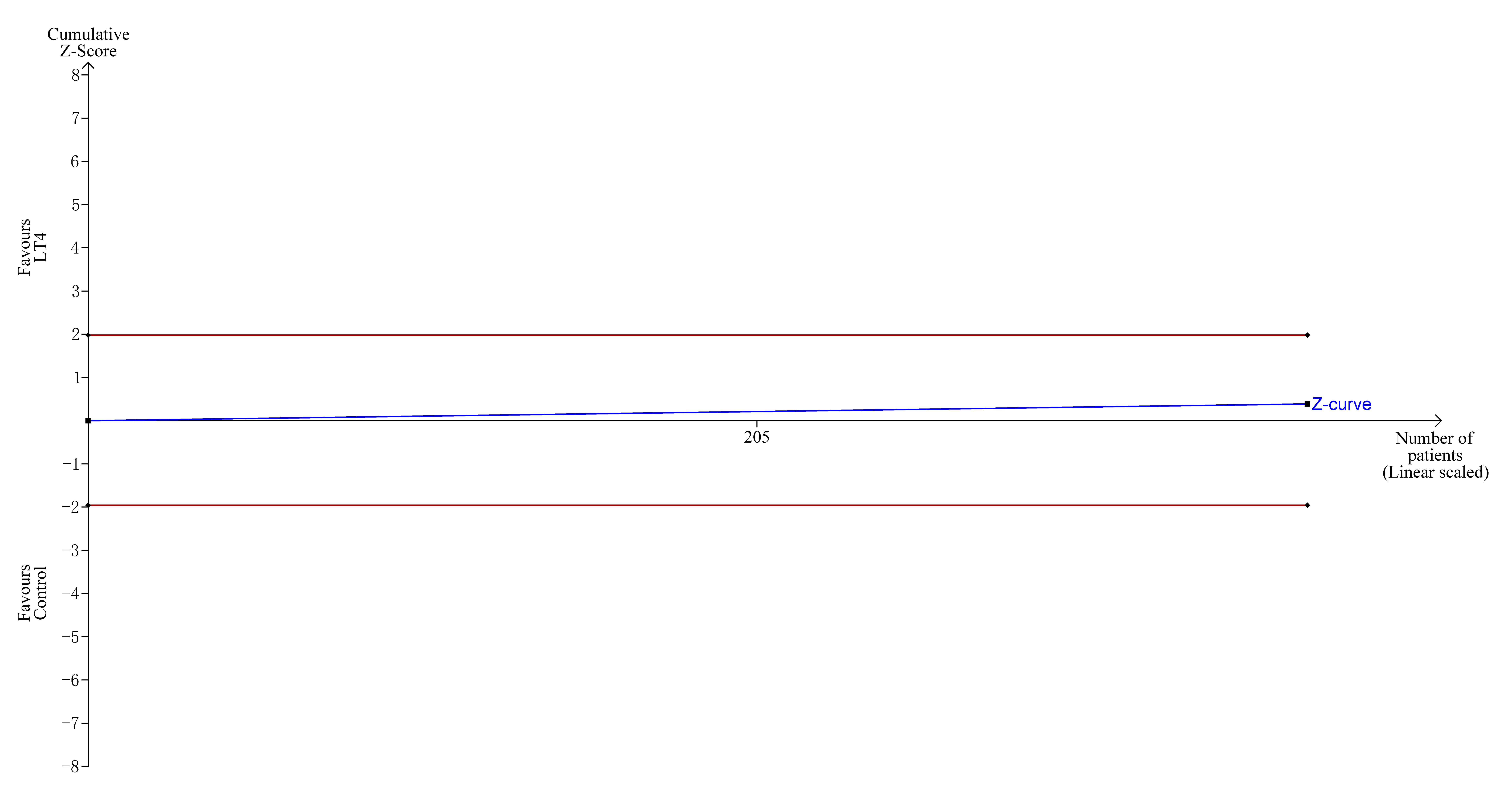
**Supplementary Figure 3. Trial sequential analysis of gestational age at delivery.** The risk of type Ⅰ error was set at 5% with a power of 80%. The variance was calculated from the data obtained from the included trials.


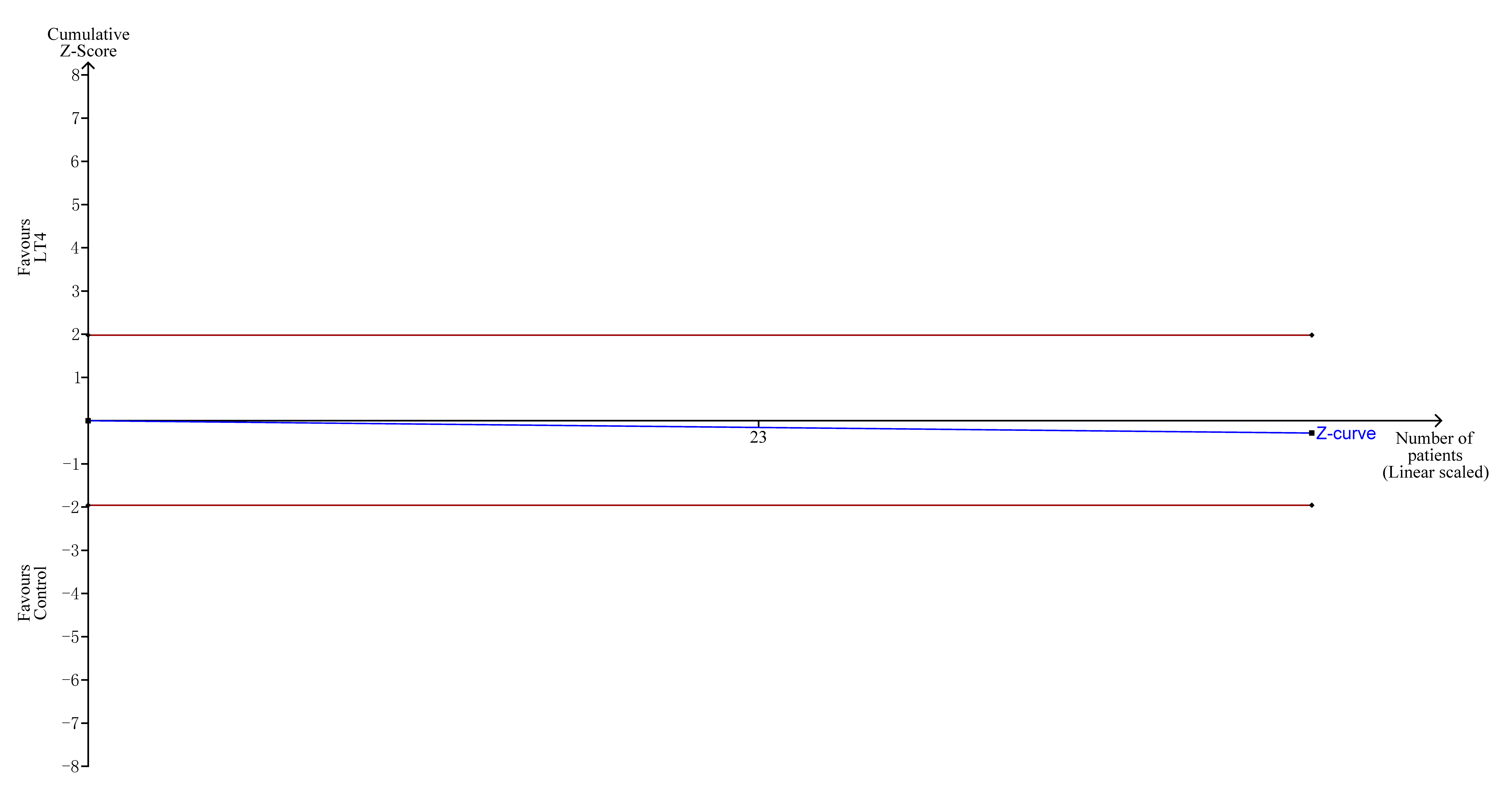


**Supplementary Figure 4. Trial sequential analysis of neonatal admission.** The risk of type Ⅰ error was set at 5% with a power of 80%. The variance was calculated from the data obtained from the included trials.

## Supplementary Tables

### Supplementary Table 1. Search strategy in Medline (Ovid).

| **#1** | exp pregnancy/ or (pregnanc* or pregnant or gestation).ti,ab,kf |
| --- | --- |
| **#2** | exp thyroid/ and exp antibodies/ |
| **#3** | (thyroid and autoimmun*).ti,ab,kf. |
| **#4** | (thyroid autoimmune antibod* or TPO-Ab* or TPOAb or "Thyroid peroxidase antibod*" or "thyroperoxidase" or thyroglobulin antibod* or Tg-Ab* or TgAb).ti,ab,kf. |
| **#5** | #2 OR #3 OR #4 |
| **#6** | (levothyroxine or euthyrox or LT4).ti,ab,kf or Thyroxine/ |
| **#7** | #1 AND #5 AND #6 |

### Supplementary Table 2. Risk of bias for RCTs

| **Study** | **Randomization process** | **Deviations from intended interventions** | **Mising outcome data** | **Measurement of the outcome** | **Selection of the reported result** | **Overall Bias** |
| --- | --- | --- | --- | --- | --- | --- |
| Nazarpour 2017 | high | some concerns | low | low | low | high |
| Negro 2005 | low | low | low | low | some concerns | some concerns |
| Negro 2006 | some concerns | high | low | high | some concerns | high |
| Negro 2016 | low | high | low | high | low | high |
| Dhillon‑Smith 2019 | low | low | low | low | low | low |
| Wang 2017 | some concerns | some concerns | low | low | low | some concerns |

### Supplementary Table 3. Risk of bias for cohort study

| **Study** | **Selection** | | | | **Comparability** | **Exposure** | | | **Score** | **Quality** |
| --- | --- | --- | --- | --- | --- | --- | --- | --- | --- | --- |
|  | **Representativ-eness of the exposed cohort** | **Selection of the non-exposed cohort** | **Ascertainment of exposure** | **Demonstration that outcome of interest was not present at start of study** | **Comparability of cohorts on the basis of the design or analysis** | **Assessment of outcome** | **Was follow-up long enough for outcomes to occur** | **Adequacy of follow-up of cohorts** |  |  |
|  |  |  |  |  |  |  |  |  |  |  |
| Revelli 2009 | 0 | 1 | 0 | 1 | 0 | 0 | 1 | 0 | 3 | Low |
| Lepoutre 2012 | 0 | 1 | 0 | 1 | 0 | 0 | 1 | 0 | 3 | Low |
| Alessandro 2021 | 0 | 1 | 0 | 1 | 0 | 0 | 1 | 0 | 3 | Low |
| Mohammad 2012 | 0 | 0 | 0 | 1 | 0 | 0 | 1 | 0 | 2 | Low |
| Tsunemi A 2021 | 0 | 1 | 0 | 1 | 0 | 0 | 1 | 1 | 4 | Moderate |

### Supplementary Table 4. Grade evidence profile for each outcome.

| **Quality assessment** | | | | | | | **No of patients** | | **Effect** | | **Quality** | **Importance** |
| --- | --- | --- | --- | --- | --- | --- | --- | --- | --- | --- | --- | --- |
|  |  |  |  |  |  |  |  |  |  |  |  |  |
| **No of studies** | **Design** | **Risk of bias** | **Inconsistency** | **Indirectness** | **Imprecision** | **Other considerations** | **LT4** | **Control** | **Relative (95% CI)** | **Absolute** |  |  |
| **Miscarriage** | | | | | | | | | | | | |
| 5 | randomised trials | no serious risk of bias | no serious inconsistency | no serious indirectness | serious^1^ | none | 119/652  (18.3%) | 141/661  (21.3%) | RR 0.85 (0.69 to 1.05) | 32 fewer per 1000 (from 66 fewer to 11 more) | ⊕⊕⊕O MODERATE | CRITICAL |
|  |  |  |  |  |  |  |  | 14.9% |  | 22 fewer per 1000 (from 46 fewer to 7 more) |  |  |
| 1 | observational studies | no serious risk of bias | no serious inconsistency | no serious indirectness | very serious^1^ | reporting bias^2^ | 3/13  (23.1%) | 1/6  (16.7%) | OR 1.5 (0.12 to 18.36) | 64 more per 1000 (from 143 fewer to 619 more) | ⊕OOO VERY LOW | CRITICAL |
|  |  |  |  |  |  |  |  |  |  | 64 more per 1000 (from 144 fewer to 619 more) |  |  |
| **Preterm birth** | | | | | | | | | | | | |
| 5 | randomised trials | no serious risk of bias | no serious inconsistency | no serious indirectness | serious^1^ | none | 67/634  (10.6%) | 86/648  (13.3%) | RR 0.8 (0.59 to 1.08) | 27 fewer per 1000 (from 54 fewer to 11 more) | ⊕⊕⊕O MODERATE | CRITICAL |
|  |  |  |  |  |  |  |  | 16.7% |  | 33 fewer per 1000 (from 68 fewer to 13 more) |  |  |
| 2 | observational studies | serious^3^ | no serious inconsistency | no serious indirectness | very serious^1^ | reporting bias^2^ | 9/84  (10.7%) | 4/50  (8%) | OR 0.95 (0.24 to 3.79) | 4 fewer per 1000 (from 60 fewer to 168 more) | ⊕OOO VERY LOW | CRITICAL |
|  |  |  |  |  |  |  |  | 4.3% |  | 2 fewer per 1000 (from 32 fewer to 103 more) |  |  |
| **Preeclampsia** | | | | | | | | | | | | |
| 1 | randomised trials | serious^4^ | no serious inconsistency | no serious indirectness | very serious^1^ | reporting bias^2^ | 2/57  (3.5%) | 3/58  (5.2%) | RR 0.68 (0.12 to 3.91) | 17 fewer per 1000 (from 46 fewer to 151 more) | ⊕OOO VERY LOW | IMPORTANT |
|  |  |  |  |  |  |  |  | 5.2% |  | 17 fewer per 1000 (from 46 fewer to 151 more) |  |  |
| **Gestational age at delivery (Better indicated by lower values)** | | | | | | | | | | | | |
| 1 | randomised trials | no serious risk of bias | no serious inconsistency | no serious indirectness | no serious imprecision | reporting bias^2^ | 186 | 188 | - | MD 0.1 lower (0.61 lower to 0.41 higher) | ⊕⊕⊕O MODERATE | IMPORTANT |
| **Neonatal admission** | | | | | | | | | | | | |
| 1 | randomised trials | serious^4^ | no serious inconsistency | no serious indirectness | very serious^1^ | reporting bias^2^ | 2/18  (11.1%) | 2/24  (8.3%) | RR 1.33 (0.21 to 8.58) | 28 more per 1000 (from 66 fewer to 632 more) | ⊕OOO VERY LOW | IMPORTANT |
|  |  |  |  |  |  |  |  | 8.3% |  | 27 more per 1000 (from 66 fewer to 629 more) |  |  |
| **Birth weight (Better indicated by lower values)** | | | | | | | | | | | | |
| 1 | randomised trials | no serious risk of bias | no serious inconsistency | no serious indirectness | no serious imprecision | reporting bias^2^ | 187 | 188 | - | MD 36 lower (170.41 lower to 98.41 higher) | ⊕⊕⊕O MODERATE | IMPORTANT |
| **Placenta abruption** | | | | | | | | | | | | |
| 1 | randomised trials | serious^4^ | no serious inconsistency | no serious indirectness | very serious^1^ | reporting bias^2^ | 0/57  (0%) | 1/58  (1.7%) | OR 0.14 (0 to 6.94) | 15 fewer per 1000 (from 17 fewer to 91 more) | ⊕OOO VERY LOW | IMPORTANT |
|  |  |  |  |  |  |  |  | 1.7% |  | 15 fewer per 1000 (from 17 fewer to 90 more) |  |  |
| 1 | observational studies | serious^4^ | no serious inconsistency | no serious indirectness | very serious^1^ | reporting bias^2^ | 1/49  (2%) | 0/47  (0%) | OR 7.09 (0.14 to 357.8) | - | ⊕OOO VERY LOW | IMPORTANT |
|  |  |  |  |  |  |  |  | 0% |  | - |  |  |

LT4, Levothyroxine; CI, confidence interval; RR, risk ratio; OR, odds ratio

^1^ 95% confidence interval was wide
^2^ The number of included studies was small and the sample size was small

^3^ All included studies had a high risk of bias
^4^ Only one study was included and the risk of bias was high
